# *Sinomenium acutum* Modulates Platelet Aggregation and Thrombus Formation by Regulating the Glycoprotein VI-Mediated Signalosome in Mice

**DOI:** 10.3390/ph17010006

**Published:** 2023-12-20

**Authors:** Yeon-Ji Kim, Tae In Kim, Ami Lee, Kyungho Kim, Youn-Hwan Hwang

**Affiliations:** 1 Korean Medicine-Application Center, Korea Institute of Oriental Medicine, Daegu 41062, Republic of Korea; yjikim@kiom.re.kr (Y.-J.K.); tikim@kiom.re.kr (T.I.K.); 2 Herbal Medicine Research Division, Korea Institution of Oriental Medicine, Daejeon 34054, Republic of Korea; dmb01367@kiom.re.kr; 3 Korean Convergence Medical Science Major, KIOM School, University of Science & Technology (UST), Daejeon 34054, Republic of Korea

**Keywords:** *Sinomenium acutum*, antiplatelet, thrombosis, collagen, glycoprotein VI

## Abstract

*Sinomenium acutum* (SA) has long been used as a traditional medicine in China, Japan, and Korea to treat a wide range of diseases. It has been traditionally used to ameliorate inflammation and improve blood circulation. However, its role in platelet activation has not been thoroughly investigated. Hence, we conducted this study to assess the potential inhibitory effect of SA on platelet aggregation and thrombus formation. The antiplatelet activities of SA were evaluated by assessing platelet aggregation, granular secretion, intracellular Ca^2+^ mobilization, and the Glycoprotein (GP) VI-mediated signalosome. The thrombosis and bleeding time assays were used to investigate the effect of SA (orally administered at 50 and 100 mg/kg for seven days) in mice. SA treatment at concentrations of 50, 100, and 200 μg/mL significantly reduced GPVI-mediated platelet aggregation, granular secretion, and intracellular Ca^2+^ mobilization. Further biochemical studies revealed that SA inhibited spleen tyrosine kinase, phospholipase Cγ2, phosphatidylinositol 3-kinase, and AKT phosphorylation. Interestingly, oral administration of SA efficiently ameliorated FeCl_3_-induced arterial thrombus formation without prolonging the tail bleeding time. These findings suggest that SA has beneficial effects in thrombosis and hemostasis. Therefore, SA holds promise as an effective therapeutic agent for the treatment of thrombotic diseases.

## 1. Introduction

Platelets are small, anucleate cell fragments that are essential for blood clotting and preventing bleeding from injuries. Unwanted platelet activation, however, is a significant contributor to the development and progression of cardiovascular disease. Once activated, platelets rapidly adhere to immobilized adhesive proteins such as von Willebrand factor and collagen, triggering platelet activation and aggregation, leading to the formation of platelet-rich thrombi. These thrombi can obstruct arteries and reduce arterial blood flow, thereby causing various diseases, including hypertension, lower-extremity deep venous thrombosis, atrial fibrillation, infective endocarditis, and heart failure [1,2].

Glycoprotein VI (GPVI) is a crucial platelet surface receptor involved in platelet function and activation. When platelets come into contact with exposed collagen, which is found in damaged blood vessel walls, a signaling cascade is initiated, resulting in platelet activation and aggregation. GPVI binds to collagen, recruits, and associates with another platelet receptor known as Fc receptor γ-chain [3]. The FcRγ-chain is essential for GPVI signaling and is also required for the expression of GPVI on the platelet surface. When GPVI forms a dimer, Src family kinases such as Fyn and Lyn phosphorylate the tyrosine residue of the FcRγ on its motif. This subsequently initiates a spleen tyrosine kinase (Syk)-dependent signaling cascade, leading to the recruitment of the linker of activated T cells (LAT) signalosome involving phospholipase C (PLC) γ2 and phosphoinositide-3 kinase (PI3K) [4,5,6]. The tyrosine phosphorylation-based activation of PLCγ2 eventually leads to intracellular Ca^2+^ accumulation, resulting in platelet activation, the release of granules, shape change, and the formation of platelet aggregates [7,8]. The activation of GPVI-mediated platelet function also triggers the release of various soluble factors, such as adenosine diphosphate (ADP) and thromboxane A2, which further enhance platelet activation and aggregation [3]. Dysfunction or abnormalities in GPVI signaling profoundly affect platelet function and hemostasis. For instance, defects in GPVI signaling pathways can lead to bleeding disorders, while excessive or inappropriate GPVI activation can contribute to thrombotic conditions such as arterial thrombosis [9]. Ongoing research on GPVI and its role in platelet function has identified the GPVI signaling pathway as a pivotal focus in the development of antiplatelet drugs.

*Sinomenium acutum* (Thumb.) Rehd. et Wils. (Menispermaceae, SA) has long been used as a traditional medicine in China, Japan, and Korea for the treatment of various diseases. It contains an alkaloid called sinomenine, which has received significant research attention for its potential anti-inflammatory, immunosuppressive, and analgesic properties [10,11]. As a result, SA has been used to alleviate pain and reduce inflammation associated with conditions like arthritis, rheumatism, and joint disorders. Furthermore, it has been used to treat fever, skin rashes, and digestive disorders [10,11]. However, the scientific information on SA remains unclear. Therefore, we conducted this study to evaluate the antiplatelet effect of SA on the process of thrombotic diseases using platelet aggregation and platelet-thrombus formation models.

The present study has demonstrated that the ethanol extract of SA has the ability to inhibit thrombus formation in vivo and in vitro, particularly by inhibiting platelet activation and aggregation induced by collagen and collagen-related peptide (CRP). In a mouse model of ferric chloride (FeCl_3_)-induced thrombosis, SA has been found to play a crucial role in collagen-induced platelet-thrombus formation. Notably, the administration of SA did not significantly increase the tail bleeding time compared to the control mice. These findings highlight the potential of SA to exert antiplatelet and antithrombotic effects without affecting hemostasis. Hence, SA holds great promise as an effective therapeutic agent for the treatment of thrombotic diseases.

## 2. Results

### 2.1. SA Suppresses GPVI-Activated Platelet Aggregation and ATP Secretion

**Figure 1:**
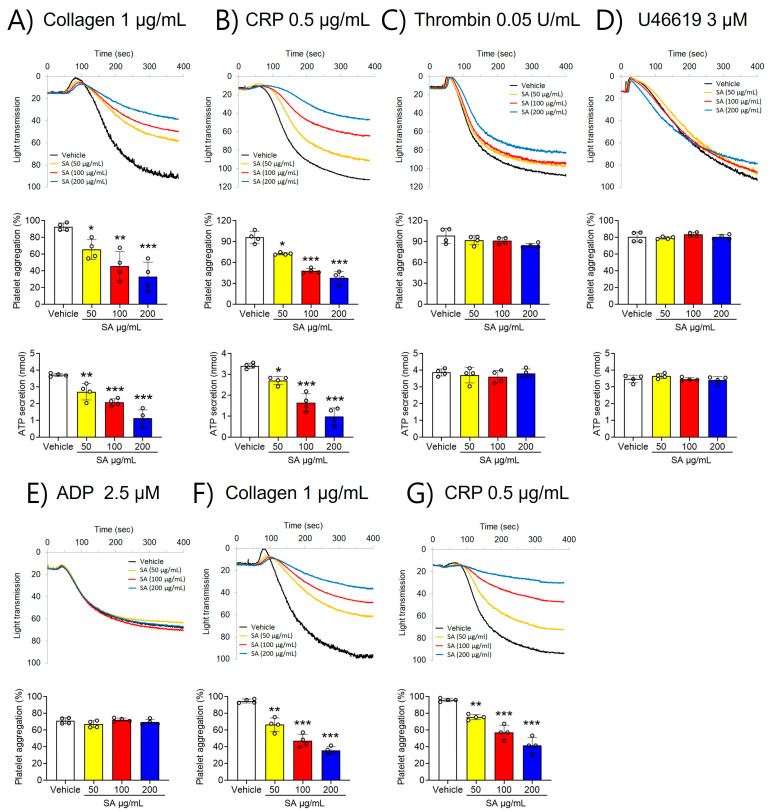
The suppressing effect of *Sinomenium acutum* (SA) on platelet aggregation and adenosine triphosphate (ATP) secretion activated by different agonists. Platelets were preincubated with the different characteristics of the solutions of SA (50, 100, and 200 µg/mL) for 10 min at 37 °C. Subsequently, they were induced by 1 µg/mL collagen (**A**), 0.5 µg/mL collagen-related peptide (CRP) (**B**), 0.05 U/mL thrombin (**C**), 3 µM U46619 (**D**), and 2.5 µM adenosine diphosphate (ADP) (**E**). In ATP secretion, cleansed platelets’ surfaces were prepared with the various concentrations of SA (50, 100, and 200 µg/mL) for 10 min at 37 °C. After the addition of the luciferin/luciferase reagent, platelets were activated with various agonists, then ATP secretion was assessed with a luminometer. PRP was preincubated with either 0.01% DMSO, or one of three concentrations of SA (50, 100, or 200 µg/mL) for 10 min at 37 °C. They were then stimulated with 1 µg/mL collagen (**F**) or 0.5 µg/mL CRP (**G**). The data are shown as means ± standard deviations (*n* = 4). *, *p* < 0.05; **, *p* < 0.01; and ***, *p* < 0.001.

To investigate the role of SA in regulating platelet function, platelet aggregation was first assessed. Our findings revealed that stimulation with various agonists, including collagen (1 µg/mL), CRP (0.5 μg/mL), thrombin (0.05 U/mL), U46619 (3 µM), and ADP (2.5 µM), significantly elevated platelet aggregation (Figure 1). However, compared to the vehicle control, SA-treated platelets showed less aggregation stimulated by collagen (≤1 μg/mL) and CRP (≤0.5 μg/mL) (Figure 1A,B). In contrast, thrombin (≤0.05 U/mL), U46619 (≤3 µM), and ADP (≤2.5 µM) did not elicit such an effect (Figure 1C–E). Furthermore, higher concentrations of collagen (≥3 µg/mL) and CRP (≥2 µg/mL) invalidated the observed defects in SA-treated platelets (Figure S1). To further estimate the effect of SA on platelet function, ATP secretion was assessed. Our findings demonstrated that SA treatment at concentrations of 50, 100, and 200 µg/mL caused a significant decrease in ATP secretion facilitated by collagen (≤1 μg/mL) and CRP (≤0.5 μg/mL) compared to the vehicle control (Figure 1A,B). However, stimulation with thrombin (0.05 U/mL) or U46619 (3 µM) did not yield comparable results (Figure 1C,D). The effect of SA on platelet activation using PRP was also investigated in comparison with that in washed platelets. We observed that collagen (1 µg/mL)- or CRP (1 µg/mL)-stimulated platelet aggregation in PRP was diminished by SA in a concentration-dependent manner similar to the effects of washing platelets (Figure 1F,G). These results suggest that plasma proteins do not influence the effects of SA on platelet aggregation. These findings suggest that SA plays an important role in GPVI-mediated platelet aggregation and ATP secretion.

### 2.2. SA Is Actively Involved in P-Selectin Exposure, αIIbβ3 Integrin Activation, and Ca^2+^ Mobilization after CRP Induction

**Figure 2:**
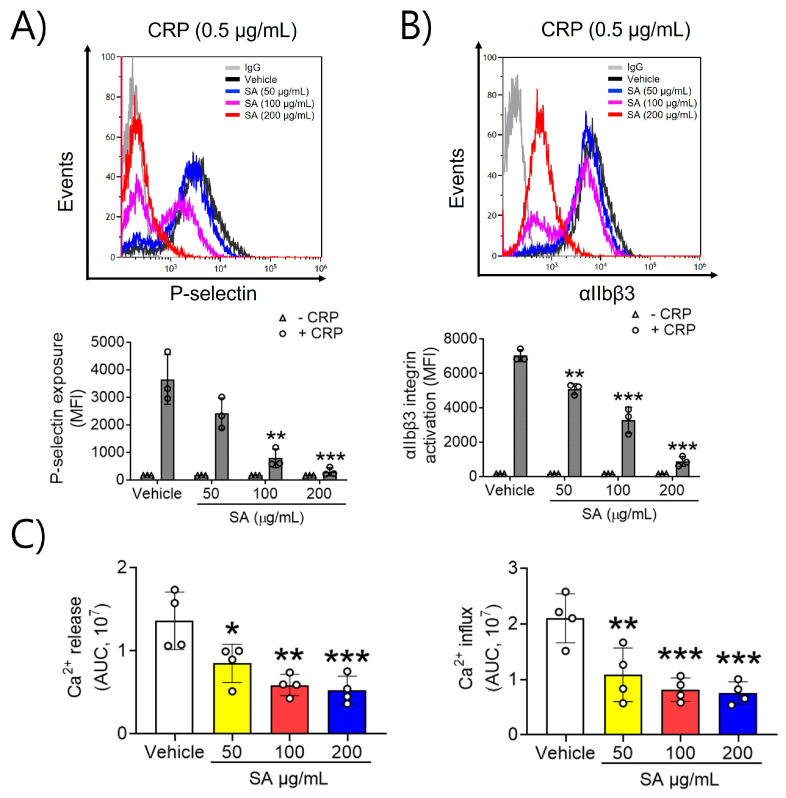
The preventive effect of *Sinomenium acutum* (SA) on P-selectin exposure, αIIbβ3 integrin stimulation, and Ca^2+^ mobilization. The binding of anti-P-selectin (**A**) and anti-activated αIIbβ3 (JON/A) antibodies (**B**) to platelets was measured as the ratio of the geometric mean fluorescence intensity value of antibodies to that of the control immunoglobulin G. The quantitative data are presented as means ± standard deviations (*n* = 3). In a Ca^2+^ mobilization assay, mouse platelets were incubated with a calcium-sensitive dye for 30 min at 37 °C in the dark, and then platelets were induced using 0.5 µg/mL collagen-related peptide (CRP) for 10 min. Intracellular Ca^2+^ secretion and flow (**C**) were evaluated and quantified with the area under the curve (arbitrary units). The quantitative data are demonstrated as means ± standard deviations (*n* = 4). *, *p* < 0.05; **, *p* < 0.01; and ***, *p* < 0.001.

To evaluate the effects of SA on platelet activation, P-selectin exposure and αIIbβ3 integrin activation, which are key processes in the positive feedback cycle of platelet activation, were assessed. In a dose-dependent manner, SA at concentrations of 50, 100, and 200 µg/mL significantly reduced P-selectin exposure and lowered αIIbβ3 integrin activation upon CRP (0.5 μg/mL) stimulation (Figure 2A,B). These findings indicate that SA effectively inhibits GPVI-mediated platelet granular secretion and αIIbβ3 integrin activation. Next, we examined the mechanism explaining the association between SA treatment and platelet activation, along with the GPVI-stimulated increase in intracellular Ca^2+^. Increases in intracellular Ca^2+^ arise through either Ca^2+^ release from intracellular Ca^2+^ stores or influx across the plasma membrane [12]. Our findings demonstrated a significant increase in intracellular Ca^2+^ release and inflow upon stimulation with CRP (0.5 μg/mL). Moreover, when platelets were pretreated with SA (50, 100, and 200 µg/mL) prior to CRP stimulation, Ca^2+^ mobilization was inhibited in a dose-dependent manner (Figure 2C). These findings indicate that SA can modulate CRP-triggered intracellular Ca^2+^ release and influx.

### 2.3. SA Is Essential in Regulating Syk, PLCγ2, PI3K, AKT, and ERK Phosphorylation Following CRP Activation

**Figure 3:**
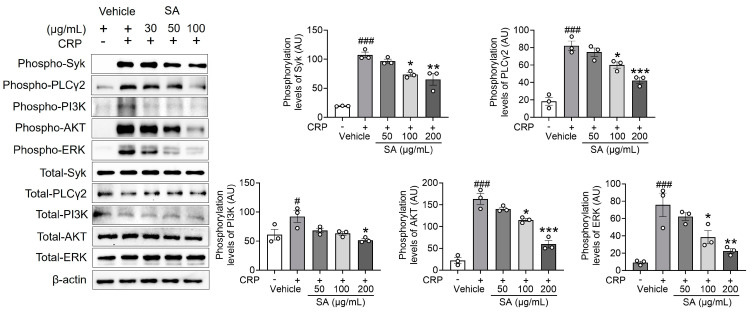
*Sinomenium acutum* (SA) extract regulates the phosphorylation of spleen tyrosine kinase (Syk), phospholipase Cγ2 (PLCγ2), phosphatidylinositol 3-kinase (PI3K), AKT, and ERK after collagen-related peptide (CRP) stimulation. Mouse platelets were pretreated with various concentrations of SA (50, 100, and 200 µg/mL) and stimulated with 0.5 µg/mL CRP. Equal amounts (30 µg) of cell lysate protein were immunoblotted to determine specific Syk, PLCγ2, PI3K, AKT, and ERK phosphorylation inhibition. The results are shown as blots and graphs. The data are presented as means ± standard deviations (*n* = 3). ^#^, *p* < 0.05 and ^###^, *p* < 0.001 versus the vehicle control (unstimulated) after Student’s *t*-test. *, *p* < 0.05; **, *p* < 0.01; and ***, *p* < 0.001 versus the vehicle control (stimulated) after analysis of variance and Tukey’s test.

The reaction to GPVI-specific agonists involves a substantial enhancement of Ca^2+^ signaling through the phosphorylation of Syk, PLCγ2, and PI3K signaling molecules [13], which, in turn, monitors platelet activation. Since SA treatment is important for the following Ca^2+^ mobilization, the effect of SA treatment on regulating Syk, PLCγ2, and PI3K phosphorylation was explored. Our findings demonstrate that SA treatment, compared to the control group, significantly diminished the phosphorylation of Syk, PLCγ2, and PI3K, as well as the downstream kinase AKT and mitogen-activated protein kinases (MAPKs), following CRP induction (Figure 3). These findings indicate that SA plays an essential role in regulating Syk, PLCγ2, PI3K, AKT, and extracellular signal-regulated kinase (ERK) phosphorylation without displaying selectivity toward specific signal transduction pathways.

### 2.4. SA prevents In Vivo Thrombosis, While Having No Impact on Hemostasis

**Figure 4:**
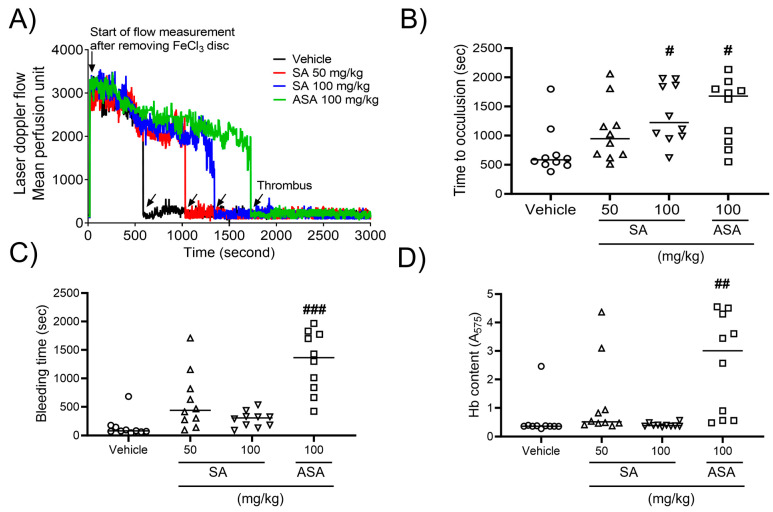
*Sinomenium acutum* (SA) delays FeCl_3_-induced arterial thrombus formation without causing bleeding. FeCl_3_-activated arterial thrombus formation was carried out, as shown in Methods. After oral administration of 0.5% low-viscosity carboxymethylcellulose (CMC) and/or SA (50 and 100 mg/kg, body weight) or acetylsalicylic acid (ASA; 100 mg/kg, body weight) once a day for seven days (**A**,**B**), 10% FeCl_3_ was injected into the mouse carotid artery for two minutes, and blood flow traces were controlled until stable occlusion was achieved. In the bleeding time assay, the tails of mice were removed, and the bleeding time (**C**) was monitored, as demonstrated in Methods. (**D**) Blood loss during the bleeding time assay was measured by assessing the absorbance at 575 nm for hemoglobin (Hb). The horizontal bars illustrate the median occlusion time (*n* = 10). ^#^, *p* < 0.05, ^##^, *p* < 0.01 and ^###^, *p* < 0.001 versus the vehicle control after Student’s *t*-test.

To explore the influence of SA on arterial thrombus generation, a mouse model of FeCl_3_-promoted carotid artery thrombosis was developed. Thrombus formation was assessed using a 10% (460 mM) FeCl_3_ solution. After FeCl_3_ application, carotid occlusion was observed at a mean time of 9.73 ± 2.23 min in the control group. However, SA therapy increased the carotid occlusion time to 15.78 ± 2.72 min at a concentration of 50 mg/kg BW or 20.83 ± 2.64 min at a concentration of 100 mg/kg BW compared to that in the positive control (27.98 ± 2.91 min) at an ASA concentration of 100 mg/kg BW (Figure 4A,B). To further evaluate the impact of oral SA administration on hemostatic function, the tail bleeding time was assessed as the timepoint of ceased bleeding after tail amputation. Our findings demonstrated no statistically significant differences in the value of this parameter between two studied groups (Figure 4C). Furthermore, the volume of blood collected from an amputation site, stratified according to hemoglobin content, did not differ between the groups. However, oral administration of ASA at a dose of 100 mg/kg significantly elevated the bleeding time and increased hemoglobin levels (Figure 4D). These findings indicate that SA plays an important role in arterial thrombosis in vivo, while not interfering with the processes of hemostasis.

### 2.5. Phytochemical Profiling of SA

**Figure 5:**
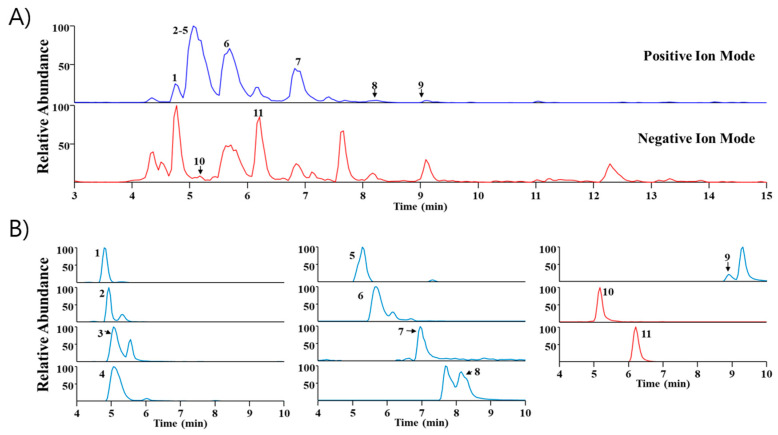
Ultra-high-performance liquid chromatography–tandem mass spectrometry examination of *Sinomenium acutum*. (**A**) Base peak chromatogram of *Sinomenium acutum* in positive and negative ion mode. (**B**) Elicit ion chromatogram of *Sinomenium acutum*. 1, higenamine; 2, acutumidine; 3, acutumine; 4, sinomenine; 5, gelsemine; 6, magnoflorine; 7, scopoletin; 8, columbamine or jatrorrhizine; 9, palmatine; 10, syringin; 11, eleutheroside E.

UHPLC-MS/MS analysis is widely utilized because it has high sensitivity and resolution and facilitates the systematic profiling of active components presents within plants [14,15]. In this study, *Sinomenium acutum* extract was analyzed via UHPLC-MS/MS to identify major components. Through a comparison of the retention time (Rt) and mass spectra with reference standards, we identified eleven phytochemicals predominantly present in the extract. These include higenamine, acutumidine, acutumine, sinomenine, gelsemine, magnoflorine, scopoletin, columbamine (or jatrorrhizine), palmatine, syringin, and eleutheroside E, all of which are found in *Sinomenium acutum* (Table 1). Figure 5 illustrates the base peak chromatograms and the extracted ion chromatograms of these identified phytochemicals in *Sinomenium acutum*.

## 3. Discussion

For this study, we evaluated the effects of SA ethanol extract on platelet function including antithrombotic effects. Our findings revealed that SA exhibited a protective effect against platelet aggregation and activation and improved platelet-thrombus formation via the GPVI-mediated platelet signalosome. Previous studies have primarily focused on the antioxidant, anti-inflammatory, analgesic, anti-allergic, immunosuppressive, anti-tumor, liver-protective, and other effects of SA. Clinical use of SA therapy has mainly been documented in relation to rheumatoid arthritis, ankylosing spondylitis, and other diseases [10,11]. However, this study is the first to describe the antiplatelet effect of SA on GPVI-mediated platelet aggregation and thrombus formation.

To investigate the underlying mechanism, we investigated GPVI-mediated intracellular Ca^2+^ mobilization, granular secretion, and fibrinogen binding to integrin αIIbβ3 (JON/A). Our findings demonstrated that SA extract markedly inhibited intracellular Ca^2+^ activation, ATP secretion, P-selectin exposure, and integrin αIIbβ3 stimulation. Targeting the collagen receptor GPVI has been shown to be an effective approach to reducing thrombosis while maintaining hemostasis [18]. Furthermore, clinical trials investigating the humanized anti-GPVI Fab fragment (ACT01) and the dimeric GPVI-Fc fusion protein (Revacept) demonstrated their inhibitory effects on the interaction between platelets and collagen without affecting general hemostasis [19,20]. These findings suggest that targeting GPVI could be a promising approach for antithrombotic treatment. In the present study, both the biochemical and in vivo results demonstrated that SA treatment could effectively prevent GPVI-mediated platelet aggregation and thrombus formation without increasing the risk of bleeding. The inhibitory effects of SA extract on platelet aggregation, activation, degranulation, and thrombus formation might be based on the inhibitory effects of the GPVI-mediated signaling pathway during cell activation.

The binding of the GPVI to immobilized collagen initiates the adhesion of flowing platelets to the subendothelial extracellular matrix and, thereby, enables the signaling pathway, which is initiated by the Src-family-kinase-mediated phosphorylation of tyrosine residues associated with the immunoreceptor tyrosine-based activation motif containing FcRγ chains [3]. Subsequently, phospholipase Cγ2 (PLCγ2) is activated by the signaling cascades involved in the recruitment and activation of spleen tyrosine kinase (Syk), the SH2 domain-containing leukocyte protein of 76 kDa (SLP-76), Proto-oncogene vav (Vav1), phosphatidylinositol 3-kinase (PI3K), and Bruton’s tyrosine kinase (Btk). The tyrosine phosphorylation-based activation of PLCγ2 eventually leads to intracellular Ca^2+^ accumulation, a marker of platelet activation and thrombus formation [7,8]. We observed that SA extract significantly inhibited the increase in phosphorylation of Syk, PLCγ2, PI3K, and AKT induced by CRP [21,22,23]. It has been demonstrated that the phosphorylation of PI3K and AKT is highly expressed in platelets, and these are key signaling pathways for GPVI downstream activation induced by collagen [24,25,26]. The PI3K and AKT pathways are downstream of Src family kinases and are the integral part of platelet activation by influencing intracellular Ca^2+^ mobilization, granular secretion, and platelet aggregation [25,26,27]. Thus, our findings suggest that the inhibitory effects of SA extract on the phosphorylation of Syk, PLCγ2, PI3K, and AKT pathways comprise an efficient and safe antiplatelet and antithrombotic strategy for GPVI-mediated platelet functions.

The FeCl_3_-promoted vascular injury model is commonly used to assess antithrombotic activity and has been well established for evaluating in vivo antithrombotic effects [2]. Tseng et al. demonstrated that FeCl_3_ accesses the endothelium through small vesicles via an endocytic–exocytic route, resulting in complete endothelial denudation [28]. This process exposes the subendothelial matrix (collagen) and leads to arterial thrombus formation with platelet activation and fibrin formation, which can be attenuated using antiplatelet agents [29]. In our study, we found that SA treatment significantly increased vascular occlusion times in mice by suppressing platelet activation without expanding the bleeding time compared to vehicle treatment. In addition, because FeCl_3_-induced injury can disrupt the endothelium, our findings further suggest that SA is important for regulating endothelial cells in arterial thrombosis. Interestingly, SA treatment did not affect the hemostatic function at the site of tail transaction [30]. Noting that the tail bleeding time may not be a reliable analysis of platelet contribution to hemostatic function, we also observed no increased bleeding from the surgery site during the FeCl_3_-induced injury arterial thrombosis study. Additionally, depending on the activities and concentrations of herbal extracts, results might differ [31]. We also observed that SA did not differ in the coagulation parameter compared with the control group (Supplemental Table S1). Consequently, unless SA concentrations exceed threshold concentration levels, there is still a chance that SA treatment will result in a reduction in antiplatelets without anticoagulation properties. Therefore, our findings suggest that SA is a potent natural candidate for alleviating platelet-related cardiovascular diseases.

Previous studies have reported that SA inhibits inflammation by suppressing nuclear factor kappa B activation in a rat model of endotoxin-induced uveitis [32]. In addition, sinomenine, the main component of SA, has been widely used in treating autoimmune diseases, as reported by numerous pharmacological studies [10]. These findings suggest that SA may have anti-inflammatory effects that can help prevent cardiovascular diseases such as atherosclerosis and stroke. Therefore, while sinomenine could be used as a principal compound for the design of SA-based antiplatelet agents, further research is needed to fully understand its effect on future drug development.

In conclusion, our findings suggest that SA treatment may have antiplatelet and antithrombotic effects by suppressing GPVI signaling. Therefore, SA can serve as a foundation for the development of new antiplatelet drugs to treat patients with cardiovascular diseases.

## 4. Materials and Methods

### 4.1. Reagent

Human thrombin, PGE1, rhodamine-phalloidin, dimethyl sulfoxide (DMSO), ADP, fibrinogen, human fibrinogen, ferric chloride (FeCl_3_), Acetylsalicylic acid (ASA), carboxymethylcellulose (CMC), and all the reagents were purchased from Sigma (St. Louis, MO, USA). Equine tendon collagen (type I) and ATP luciferin/luciferase reagent were obtained from Chrono-log (Havertown, PA, USA). CRP was obtained from Dr Richard Farndale (Department of Biochemistry, University of Cambridge, UK). Phycoerythrin (PE)-conjugated isotype control IgGs, rat monoclonal antibodies against mouse P-selectin, activated αIIbβ3 (JON/A) were from Emfret Analytics (Eibelstadt, Germany). Antibodies against phospho-Syk at Tyr525/526 (Cat# 2710S), phospho-PLCγ2 at Tyr1217 (Cat# 3871S), phospho-PI3K p85α/β at Tyr458/p55α/γ at Tyr199 (Cat# 4228S), phospho-Akt at Ser473 (Cat# 9271S), phospho-ERK at Thr202/Tyr204 (Cat# 4337S), Total Syk (Cat# 12358S), Total PLCγ2 (Cat# 3872S), Total PI3K p85 (Cat# 4249S), Total Akt (Cat# 9272S), Total ERK (Cat# 9102S), and β-actin (Cat# 4967S) were obtained from Cell Signaling (Danvers, MA, USA). Monoclonal antibodies against phospho-integrin β3 at Tyr 759 and Total integrin β3 were obtained from Santa Cruz (Santa Cruz, CA, USA). Calcium dye (FLIPR Calcium Assay kit) was from Molecular Devices (Sunnyvale, CA, USA).

### 4.2. Herbal Medicine Preparation

The SA was received from Omniherb Co., Ltd. (Yerongcheon, Korea). To prepare the extract, the dried SA (100 g) was mixed into 1000 mL of 30% ethyl alcohol in an extractor (Gyeongseo Extractor Cosmos-600, Inchon, Korea) for 12 h. The obtained extract then underwent filtering through a 150 μm standard test sieve (Retsch, Hann, Germany) and subsequent freeze drying, resulting in a concentration of 4.3%. The lyophilized powder was then dissolved in 0.01% DMSO, finally reaching a concentration of 100 mg/mL.

### 4.3. Animals

Male mice of the wild-type C57BL/6 strain, aged 6–8 weeks and weighing 18–22 g, were received from DooYeol Biotech (Seoul, Korea) and allowed to acclimate for one week. There were a total of four random groups, with ten animals in each. They were as follows: (1) a vehicle group, receiving 0.5% low-viscosity CMC orally; (2) a low-dose SA group, with 50 mg/kg body weight (BW); (3) a high-dose SA group, administered 100 mg/kg BW; and (4) an ASA group, receiving 100 mg/kg BW. They were maintained under monitored conditions of temperature and humidity, with a 12/12 h light/dark cycle. The animals were treated in accordance with the National Animal Welfare Law of Korea, and all the performed animal experiments (reference number #23-035) were conducted in compliance with the Korea Institute of Oriental Medicine (KIOM, Daegu, Korea) Care Committee guidelines.

### 4.4. Platelet Preparation

Whole-blood samples were obtained from mice with acid-citrate-dextrose (ACD) buffer (Sigma). To collect platelet-rich plasma (PRP), the whole blood was centrifuged at 300× *g* for 20 min with no brake applied, and prostaglandin E1 (0.5 µM) was added to prevent platelet activation. The PRP was then recentrifuged at 700 g for 4 min to obtain the platelet pellet. The isolated pellet was suspended in 4-(2-hydroxyethyl)-1-piperazineethanesulfonic acid (HEPES)-Tyrode buffer (5 mM HEPES/NaOH, pH 7.3, 5 mM glucose, 136 mM NaCl, 12 mM NaHCO_3_, 2.7 mM KCl) containing 10% ACD and recentrifuged at 700 g for 5 min. The resulting final pellet was counted using a hemocytometer and adjusted to a concentration of 3 × 10^8^ platelets/mL with HEPES-Tyrode buffer [33,34,35].

### 4.5. Platelet Aggregation and ATP Secretion

The platelets were placed in modified HEPES-Tyrode buffer and then grown with either 0.01% DMSO or one of three concentrations of SA (50, 100, or 200 µg/mL) for 10 min at 37 °C. They were then activated via various agonists, including collagen (1 µg/mL), CRP (0.5 µg/mL), thrombin (0.05 U/mL), U46619 (3 µM), or ADP (2.5 µM). In some experiments, PRP was preincubated with either 0.01% DMSO or one of three concentrations of SA (50, 100, or 200 µg/mL) for 10 min at 37 °C. They were then stimulated with collagen (1 µg/mL) or CRP (0.5 µg/mL). In the case of ADP-facilitated aggregation, human FG (30 µg/mL) was added to the platelet suspension prior to ADP induction. Platelet aggregation was determined with a 4-channel platelet Lumi-aggregometer (Chrono-Log Corp., Havertown, PA, USA) at 37 °C with stirring at 1000 rpm. Platelet secretion was controlled by measuring that of ADP/ATP through the addition of luciferin/luciferase reagent (Chrono-log) to the platelet suspension [36,37].

### 4.6. Flow Cytometric Analysis

The rinsed platelets were treated with either 0.01% DMSO or one of three concentrations of SA (50, 100, or 200 µg/mL) for 10 min at 37 °C. They were then processed with 0.5 μg/mL of CRP for five minutes at 37 °C and incubated with phycoerythrin-conjugated antibodies against P-selectin or induced αIIbβ3 integrin (JON/A) for 15 min. The cells were then evaluated using flow cytometry (Gallios, Beckman Coulter, Bera, CA, USA).

### 4.7. Ca^2+^ Mobilization

The rinsed platelets (1 × 10^8^/mL) were immersed in HEPES-Tyrode buffer (pH 7.4) without CaC_l2_ and subjected to treatment with either 0.01% DMSO or one of three concentrations of SA (50, 100, or 200 µg/mL) for 10 min at 37 °C. The cells were then stained with a Ca^2+^ dye (FLIPR Calcium 5 Assay kit) for 30 min at 37 °C in the dark and then stimulated with CRP (0.2 µg/mL). Cytosolic Ca^2+^ levels were determined with a spectrofluorometer (Spectramax I3, Molecular Devices) with an excitation wavelength of 485 nm and an emission of 525 nm. Ca^2+^ mobilization was evaluated by measuring the area under the curve and expressed as a relative fluorescence unit.

### 4.8. Immunoblotting

Mouse platelets were preincubated with three different concentrations of SA (50, 100, or 200 µg/mL) for 10 min and then stimulated with 0.5 µg/mL CRP for 5 min under constant stirring at 1000 rpm in an aggregometer. To measure the phosphorylation levels of kinases, platelets were lysed in lysis buffer. The lysed platelets were then sonicated. An equal amount of protein (30 µg) was electrophoresed under reduced conditions and subsequently transferred to polyvinylidene difluoride membranes (Millipore, Billerica, MA, USA). The immunoblot was blocked with TBS-T buffer containing 5% BSA for 1 h and incubated overnight at 4 °C with different primary antibodies. After being washed 4 times for 10 min each with TBS-T buffer, the blots were probed with a Horseradish peroxidase (HRP)-conjugated secondary antibody (1:5000, Santa CruZ Biotechnology, Santa Cruz, CA, USA, sc-2004 and sc-2005) for 1 h at room temperature, and the membranes were visualized using enhanced chemiluminescence. The band density was quantified via densitometry using ImageJ software (v1.52a). Phosphorylation levels of kinases were determined by normalizing the density of antibodies against phosphorylated kinases to that of antibodies against total kinases.

### 4.9. FeCl_3_-Induced In Vivo Thrombosis and Tail Bleeding Time

Mice were administered 0.5% low-viscosity CMC or SA (50 or 100 mg/kg BW) or ASA (100 mg/kg BW) orally once a day for seven days. On the final day, the administration was performed 30 min prior to the experiments. Following the last administration, the mice were anesthetized with 2.5% isoflurane in an oxygen mixture. The left carotid artery was isolated, and a filter paper (2 mm in diameter) soaked in 10% (460 mM) FeCl_3_ was placed over the artery for two minutes. Blood flow was then monitored until 10 min after blood occlusion using a blood flowmeter (AD Instruments). For the bleeding assay, one hour after the last administration, mice were anaesthetized, and the tail bleeding assay was performed as previously described [34].

### 4.10. Phytochemical Profiling of Sinomenium acutum with Ultra-High-Performance Liquid Chromatography–Tandem Mass Spectrometry (UHPLC–MS/MS)

The phytochemical components of *Sinomenium acutum* were identified as depicted above [16,17] and compared with the reference retention time and mass spectrum. The latter included higenamine, gelsemine, magnoflorine, scopoletin, columbamine, jatrorrhizine, palmatine, syringin, and eleutheroside E, which were purchased from TargetMol (Wellesley Hills, MA, USA). UHPLC–MS/MS analysis was performed with a Dionex UltiMate 3000 system coupled with a Thermo Q-Exactive mass spectrometer (Thermo Fisher Scientific, Waltham, MA, USA). The dried powder of *Sinomenium acutum* extract was added to 100% MeOH to attain a concentration of 20 mg/mL. Chromatographic separation was performed using an Acquity BEH C18 column (100 × 2.1 mm, 1.7 µm) with 0.1% formic acid in water and acetonitrile. The gradient eluent condition for UHPLC and the details for MS/MS were incorporated, as previously shown [38]. Data acquisition and processing were carried out withXcalibur and TraceFinder 5.1 software systems (Thermo Fisher Scientific).

### 4.11. Statistical Analysis

Data analyses were implemented with GraphPad Prism 9.5.1. An analysis of variance followed by Tukey’s or Dunnett’s test was applied for the comparison of multiple groups, while Student’s *t*-test was employed for that between two groups. A *p*-value of <0.05 was regarded statistically significant.

## Figures and Tables

**Table 1:** Phytochemical components of *Sinomenium acutum* via UHPLC-MS/MS.

No	R_t_ (min)	Calculated (*m*/*z*)	Measured (*m*/*z*)	Error (ppm)	Adduct	Formula	MS/MS Fragments (*m*/*z*)	Identification
1	4.79	272.1281	272.1283	0.6464	M+H	C_16_H_17_NO_3_	255, 107, 161	Higenamine *
2	4.93	384.1208	384.1212	0.8195	M+H	C_18_H_22_ClNO_6_	305, 341	Acutumidine [16]
3	5.06	398.1365	398.1369	1.0345	M+H	C_19_H_24_ClNO_6_	305, 341	Acutumine [17]
4	5.06	330.17	330.1703	0.9331	M+H	C_19_H_23_NO_4_	-	Sinomenine [17]
5	5.29	323.1754	323.1761	2.0296	M+H	C_20_H_22_N_2_O_2_	278	Gelsemine *
6	5.69	342.17	342.1703	0.9004	M+	C_20_H_24_NO_4_	265, 297	Magnoflorine *
7	6.96	193.0495	193.0499	1.9244	M+H	C_10_H_8_O_4_	105, 133, 161	Scopoletin *
8	8.15	338.1387	338.1389	0.5175	M+	C_20_H_20_NO_4_	-	Columbamine or Jatrorrhizine *
9	8.89	352.1543	352.1546	0.6859	M+	C_21_H_22_NO_4_	336	Palmatine *
10	5.17	417.1402	417.1412	2.4101	M+HCO_2_	C_17_H_24_O_9_	149, 271	Syringin *
11	6.21	787.2666	787.2681	1.8373	M+HCO_2_	C_34_H_46_O_18_	417	Eleutheroside E *

*, The comparison with the reference values of retention time and mass spectrum. R_t_, retention time.

## Data Availability

All the data supporting the results were shown in the paper, and can be obtained from the corresponding author.

## Supplementary Materials

The following supporting information can be downloaded at: https://www.mdpi.com/article/10.3390/ph17010006/s1, Figure S1: Role of *Sinomenium acutum* (SA) in platelet aggregation following collagen and CRP stimulation. Table S1: Coagulation parameters in SA treatment.

## References

[1] Zhou Y., Zhang D., Tan P., Xian B., Jiang H., Wu Q., Huang X., Zhang P., Xiao X., Pei J. (2023). Mechanism of platelet activation and potential therapeutic effects of natural drugs. Phytomedicine.

[2] Furie B., Furie B.C. (2008). Mechanisms of thrombus formation. N. Engl. J. Med..

[3] Nieswandt B., Watson S.P. (2003). Platelet-collagen interaction: Is gpvi the central receptor?. Blood.

[4] Lowell C.A. (2011). Src-family and syk kinases in activating and inhibitory pathways in innate immune cells: Signaling cross talk. Cold Spring Harb. Perspect. Biol..

[5] Watson S.P., Asazuma N., Atkinson B., Berlanga O., Best D., Bobe R., Jarvis G., Marshall S., Snell D., Stafford M. (2001). The role of itam- and itim-coupled receptors in platelet activation by collagen. Thromb. Haemost..

[6] Dütting S., Bender M., Nieswandt B. (2012). Platelet gpvi: A target for antithrombotic therapy?!. Trends Pharmacol. Sci..

[7] Munnix I.C., Strehl A., Kuijpers M.J., Auger J.M., van der Meijden P.E., van Zandvoort M.A., oude Egbrink M.G., Nieswandt B., Heemskerk J.W. (2005). The glycoprotein vi-phospholipase cgamma2 signaling pathway controls thrombus formation induced by collagen and tissue factor in vitro and in vivo. Arterioscler. Thromb. Vasc. Biol..

[8] Varga-Szabo D., Braun A., Nieswandt B. (2009). Calcium signaling in platelets. J. Thromb. Haemost..

[9] Nurden A.T. (2019). Clinical significance of altered collagen-receptor functioning in platelets with emphasis on glycoprotein vi. Blood Rev..

[10] Ding C., Li Y., Sun Y., Wu Y., Wang F., Liu C., Zhang H., Jiang Y., Zhang D., Song X. (2022). *Sinomenium acutum*: A comprehensive review of its botany, phytochemistry, pharmacology and clinical application. Am. J. Chin. Med..

[11] Zhao X.X., Peng C., Zhang H., Qin L.P. (2012). *Sinomenium acutum*: A review of chemistry, pharmacology, pharmacokinetics, and clinical use. Pharm. Biol..

[12] Stefanini L., Roden R.C., Bergmeier W. (2009). Caldag-gefi is at the nexus of calcium-dependent platelet activation. Blood.

[13] Berlanga O., Bobe R., Becker M., Murphy G., Leduc M., Bon C., Barry F.A., Gibbins J.M., Garcia P., Frampton J. (2000). Expression of the collagen receptor glycoprotein vi during megakaryocyte differentiation. Blood.

[14] Zou Y.T., Long F., Wu C.Y., Zhou J., Zhang W., Xu J.D., Zhang Y.Q., Li S.L. (2019). A dereplication strategy for identifying triterpene acid analogues in poria cocos by comparing predicted and acquired uplc-esi-qtof-ms/ms data. Phytochem. Anal..

[15] Jang S., Lee A., Hwang Y.-H. (2022). Qualitative profiling and quantitative analysis of major constituents in jinmu-tang by uhplc-q-orbitrap-ms and uplc-tq-ms/ms. Molecules.

[16] Wang Y., Gao X., Wang J., Tang M., Yu B., Wang Z., Cao L., Chen X., Qian M., Wang S. (2022). Identification and characterization of major alkaloid from sinomenium acutum stem and their metabolites after oral administration in rat plasma, urine, bile and feces based on uplc-q-tof/ms. J. Pharm. Biomed. Anal..

[17] Huang Y.-F., He F., Wang C.-J., Xie Y., Zhang Y.-Y., Sang Z., Qiu P., Luo P., Xiao S.-Y., Li J. (2020). Discovery of chemical markers for improving the quality and safety control of *Sinomenium acutum* stem by the simultaneous determination of multiple alkaloids using uhplc-qqq-ms/ms. Sci. Rep..

[18] McFadyen J.D., Schaff M., Peter K. (2018). Current and future antiplatelet therapies: Emphasis on preserving haemostasis. Nat. Rev. Cardiol..

[19] Lebozec K., Jandrot-Perrus M., Avenard G., Favre-Bulle O., Billiald P. (2017). Design, development and characterization of act017, a humanized fab that blocks platelet’s glycoprotein vi function without causing bleeding risks. mAbs.

[20] Ungerer M., Rosport K., Bultmann A., Piechatzek R., Uhland K., Schlieper P., Gawaz M., Munch G. (2011). Novel antiplatelet drug revacept (dimeric glycoprotein vi-fc) specifically and efficiently inhibited collagen-induced platelet aggregation without affecting general hemostasis in humans. Circulation.

[21] Law D.A., Nannizzi-Alaimo L., Ministri K., Hughes P.E., Forsyth J., Turner M., Shattil S.J., Ginsberg M.H., Tybulewicz V.L., Phillips D.R. (1999). Genetic and pharmacological analyses of syk function in alphaiibbeta3 signaling in platelets. Blood.

[22] van Eeuwijk J.M., Stegner D., Lamb D.J., Kraft P., Beck S., Thielmann I., Kiefer F., Walzog B., Stoll G., Nieswandt B. (2016). The novel oral syk inhibitor, bl1002494, protects mice from arterial thrombosis and thromboinflammatory brain infarction. Arterioscler. Thromb. Vasc. Biol..

[23] Braselmann S., Taylor V., Zhao H., Wang S., Sylvain C., Baluom M., Qu K., Herlaar E., Lau A., Young C. (2006). R406, an orally available spleen tyrosine kinase inhibitor blocks fc receptor signaling and reduces immune complex-mediated inflammation. J. Pharmacol. Exp. Ther..

[24] Senis Y.A., Atkinson B.T., Pearce A.C., Wonerow P., Auger J.M., Okkenhaug K., Pearce W., Vigorito E., Vanhaesebroeck B., Turner M. (2005). Role of the p110delta pi 3-kinase in integrin and itam receptor signalling in platelets. Platelets.

[25] Lian L., Wang Y., Draznin J., Eslin D., Bennett J.S., Poncz M., Wu D., Abrams C.S. (2005). The relative role of plcbeta and pi3kgamma in platelet activation. Blood.

[26] Adam F., Kauskot A., Rosa J.P., Bryckaert M. (2008). Mitogen-activated protein kinases in hemostasis and thrombosis. J. Thromb. Haemost..

[27] Gilio K., Munnix I.C., Mangin P., Cosemans J.M., Feijge M.A., van der Meijden P.E., Olieslagers S., Chrzanowska-Wodnicka M.B., Lillian R., Schoenwaelder S. (2009). Non-redundant roles of phosphoinositide 3-kinase isoforms alpha and beta in glycoprotein vi-induced platelet signaling and thrombus formation. J. Biol. Chem..

[28] Tseng M.T., Dozier A., Haribabu B., Graham U.M. (2006). Transendothelial migration of ferric ion in fecl3 injured murine common carotid artery. Thromb. Res..

[29] Eckly A., Hechler B., Freund M., Zerr M., Cazenave J.P., Lanza F., Mangin P.H., Gachet C. (2011). Mechanisms underlying fecl3-induced arterial thrombosis. J. Thromb. Haemost..

[30] Kurz K.D., Main B.W., Sandusky G.E. (1990). Rat model of arterial thrombosis induced by ferric chloride. Thromb. Res..

[31] Chukwumah Y., Walker L., Vogler B., Verghese M. (2007). Changes in the phytochemical composition and profile of raw, boiled, and roasted peanuts. J. Agric. Food Chem..

[32] Kim T.W., Han J.M., Han Y.K., Chung H. (2018). Anti-inflammatory effects of *Sinomenium acutum* extract on endotoxin-induced uveitis in lewis rats. Int. J. Med. Sci..

[33] Kim Y.J., Kim T.I., Kim K. (2023). An optimized herbal medicine containing *Scutellaria baicalensis* Georgi, *Alisma orientale* Juzepzuk, and *Atractylodes japonica* Koidzumi has potent antiplatelet and antithrombotic activities. J. Tradit. Complement. Med..

[34] Kim T.I., Kim Y.J., Kim K. (2021). Extract of seaweed inhibits integrin αiibβ3-induced outside-in signaling and arterial thrombosis. Front. Pharmacol..

[35] Amirou A., Bnouham M., Legssyer A., Ziyyat A., Aziz M., Berrabah M., Mekhfi H. (2018). Effects of root bark extract on platelet aggregation, bleeding time, and plasmatic coagulation: And experiments. Evid.-Based Complement. Altern. Med..

[36] Endale M., Lee W.M., Kamruzzaman S.M., Kim S.D., Park J.Y., Park M.H., Park T.Y., Park H.J., Cho J.Y., Rhee M.H. (2012). Ginsenoside-rp1 inhibits platelet activation and thrombus formation via impaired glycoprotein vi signalling pathway, tyrosine phosphorylation and mapk activation. Br. J. Pharmacol..

[37] Kamruzzaman S.M., Endale M., Oh W.J., Park S.C., Kim K.S., Hong J.H., Kwak Y.S., Yun B.S., Rhee M.H. (2010). Inhibitory effects of aqueous extract on agonist-induced platelet activation and thrombus formation involves mitogen-activated protein kinases. J. Ethnopharmacol..

[38] Hwang Y.H., Jang S.A., Kim T., Ha H. (2019). Anti-osteoporotic and anti-adipogenic effects of rhus chinensis nutgalls in ovariectomized mice fed with a high-fat diet. Planta Med..

