# Supplementary material for: Sinomenium acutum Modulates Platelet Aggregation and Thrombus Formation by Regulating the Glycoprotein VI-Mediated Signalosome in Mice
Source: Pharmaceuticals (Basel). 2023 Dec 20;17(1):6. doi: 10.3390/ph17010006 (PMC10819422; doi:10.3390/ph17010006)
Supplement: Supplementary file 1 [file pharmaceuticals-17-00006-s001.zip › pharmaceuticals-2731324-supplementary.pdf]

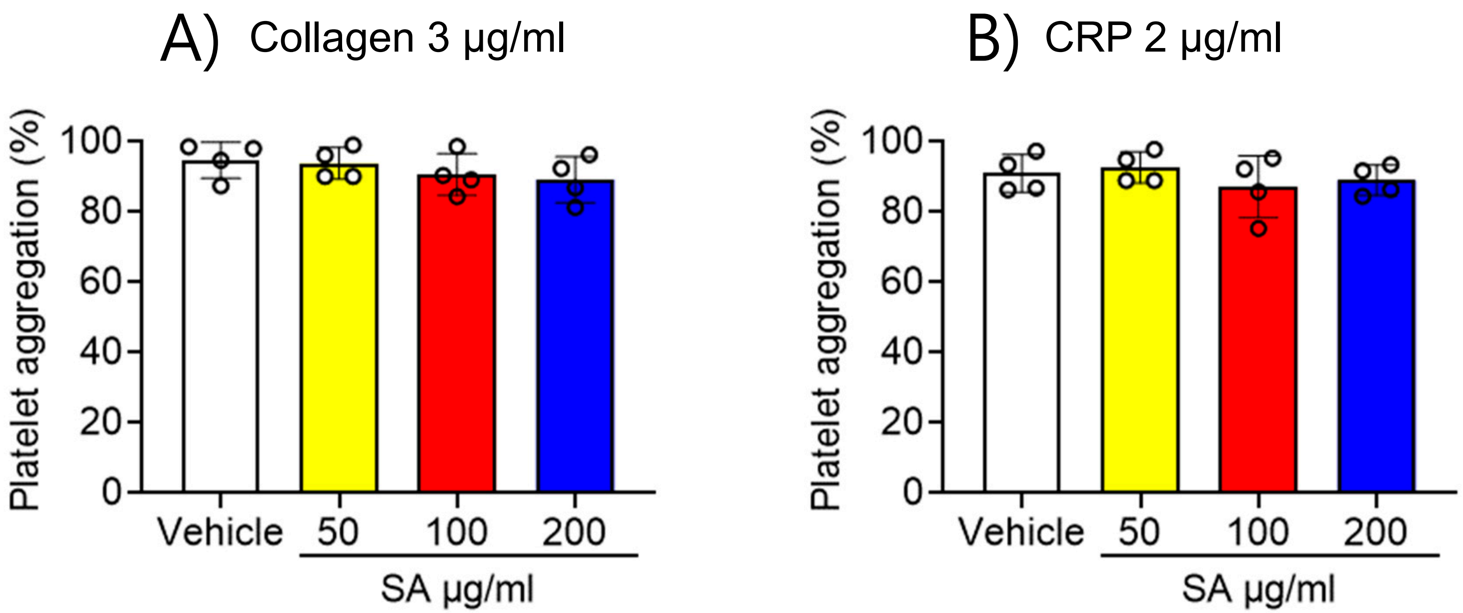

Figure. S1. Role of *Sinomenium acutum* (SA) in platelet aggregation following collagen and CRP stimulation. Washed platelets were preincubated with various concentrations of SA (50, 100, and 200 μg/mL) for 10 minutes at 37°C and then stimulated with 3 μg/ml Collagen (A) and 2 μg/ml CRP (B). Data represent the mean ± SD (n = 4).

Supplementary Table S1. Coagulation parameters in SA treatment.

	Control level	Mean ± SD
PT (Sec)	Vehicle	6.7 ± 0.24
	SA 50 mg/kg	6.7 ± 0.25
	SA 100 mg/kg	6.6 ± 0.25
aPTT (Sec)	Vehicle	37.1 ± 4.12
	SA 50 mg/kg	38.8 ± 6.26
	SA 100 mg/kg	38.6 ± 4.44

Coagulation parameters from oral administration (7 days) of the vehicle and/or SA (50 and 100 mg/kg, BW) mice were measured using a coagulation analyzer (ACL 7000, Instrumentation laboratory comp. Bedford, MA, USA). Data represent the mean ± SD (n = 3 mice per group). There was no significant difference in prothrombin time (PT) and activated partial thromboplastin time (APTT) in the SA treatment, compared to the control group. PT: prothrombin time; aPTT: activated partial thromboplastin time; Sec: second.
